# Robotic-assisted contra open resection for suspected or confirmed gallbladder cancer (ROBOCOP)

**DOI:** 10.1093/bjsopen/zrae168

**Published:** 2025-02-20

**Authors:** Hannes Wållgren, Meimene Khalil, Helena Taflin, Caroline Williamsson, Stefan Gilg, Dennis Björk, Malin Sternby Eilard, Peter Strandberg Holka, Melroy D’Souza, Linda Lundgren, Christian Cahlin, Bodil Andersson, Ernesto Sparrelid, Per Sandström, Niclas Kvarnström, Magnus Rizell, Jenny Lundmark Rystedt, Bergthor Björnsson, Christian Sturesson

**Affiliations:** Division of Surgery, Department of Clinical Science, Intervention and Technology (CLINTEC), Karolinska Institutet and Karolinska University Hospital, Stockholm, Sweden; Department of Surgery in Linköping and Department of Biomedical and Clinical Sciences, Linköping University, Linköping, Sweden; Transplant Institute, Institute of Clinical Sciences, Sahlgrenska Academy at University of Gothenburg, Sahlgrenska University Hospital, Gothenburg, Sweden; Department of Clinical Sciences Lund, Surgery, Lund University and Skåne University Hospital, Lund, Sweden; Division of Surgery, Department of Clinical Science, Intervention and Technology (CLINTEC), Karolinska Institutet and Karolinska University Hospital, Stockholm, Sweden; Department of Surgery in Linköping and Department of Biomedical and Clinical Sciences, Linköping University, Linköping, Sweden; Transplant Institute, Institute of Clinical Sciences, Sahlgrenska Academy at University of Gothenburg, Sahlgrenska University Hospital, Gothenburg, Sweden; Department of Clinical Sciences Lund, Surgery, Lund University and Skåne University Hospital, Lund, Sweden; Division of Surgery, Department of Clinical Science, Intervention and Technology (CLINTEC), Karolinska Institutet and Karolinska University Hospital, Stockholm, Sweden; Department of Surgery in Linköping and Department of Biomedical and Clinical Sciences, Linköping University, Linköping, Sweden; Transplant Institute, Institute of Clinical Sciences, Sahlgrenska Academy at University of Gothenburg, Sahlgrenska University Hospital, Gothenburg, Sweden; Department of Clinical Sciences Lund, Surgery, Lund University and Skåne University Hospital, Lund, Sweden; Division of Surgery, Department of Clinical Science, Intervention and Technology (CLINTEC), Karolinska Institutet and Karolinska University Hospital, Stockholm, Sweden; Department of Surgery in Linköping and Department of Biomedical and Clinical Sciences, Linköping University, Linköping, Sweden; Transplant Institute, Institute of Clinical Sciences, Sahlgrenska Academy at University of Gothenburg, Sahlgrenska University Hospital, Gothenburg, Sweden; Transplant Institute, Institute of Clinical Sciences, Sahlgrenska Academy at University of Gothenburg, Sahlgrenska University Hospital, Gothenburg, Sweden; Department of Clinical Sciences Lund, Surgery, Lund University and Skåne University Hospital, Lund, Sweden; Department of Surgery in Linköping and Department of Biomedical and Clinical Sciences, Linköping University, Linköping, Sweden; Division of Surgery, Department of Clinical Science, Intervention and Technology (CLINTEC), Karolinska Institutet and Karolinska University Hospital, Stockholm, Sweden

Minimally invasive surgery, including laparoscopic and robotic-assisted surgery, is gaining in popularity in the field of hepato-pancreato-biliary surgery with the aim to shorten the time to functional recovery by reducing postoperative pain and minimizing complications. Different procedures have been evaluated showing a probable benefit for minimally invasive surgery in distal pancreatectomy^1^ and liver resections^2^.

The technique of minimally invasive surgery for resection of suspected or confirmed gallbladder cancer has been studied to a limited extent. Traditionally, open surgery has been preferred over minimally invasive surgery due to the difficulty in achieving adequate lymphadenectomy and because of the complexity of liver resection. For operable patients with gallbladder cancer ≥T1b without evidence of distant spread, resection is advocated including a wedge resection of the liver comprising the gallbladder fossa or resection of liver segments 4a and 5, and dissection of regional lymph nodes (radical cholecystectomy). The extent of lymph node dissection needed for proper staging is debated, but lymphadenectomy of the hepatoduodenal ligament and retrieval of six or more lymph nodes has been suggested^3^.

Case studies have shown that robotic-assisted surgery for gallbladder cancer may have benefits such as minimizing the surgical stress on the patient, reducing duration of hospital stay^4^. Existing data suggest that the oncological outcome, as mirrored by the number of retrieved lymph nodes, is equivalent between open surgery and minimally invasive surgery^5^. As of now, evidence is of low quality.

The ROBOCOP trial is a multicentre, open-label, randomized clinical superiority trial conducted in centres experienced in robotic-assisted liver surgery. Patients with suspected or confirmed gallbladder cancer eligible for radical cholecystectomy will be randomized in a 1:1 ratio to undergo robotic-assisted or open resection within an enhanced recovery setting.

The primary endpoint is time to functional recovery (days) defined as when the patient has adequate pain control with oral analgesics only (Numerical Rating Scale <5), is independently mobile at the preoperative level, can maintain sufficient oral caloric intake (minimum of 50% required calories), has no need for intravenous fluid administration, and has no clinical signs of infection or infection treated with peroral antibiotics only.

Secondary endpoints include duration of hospital stay, resection margin, number of retrieved lymph nodes, postoperative complications, quality of life, abdominal wall complaints, and direct and indirect costs.

Robotic-assisted surgery is hypothesized to reduce time to functional recovery by 2 days compared with open surgery. With a power of 80% and *a* of 0.05, the sample size needed in each arm to test superiority is 35 patients. With an expected 25% drop-out rate, 47 patients will be randomized in each group, with a total of 94 patients to be randomized in the trial. Block randomization stratified at centre with a block size of 4 will be used.

More in-depth information of the protocol can be found in *Supplementary methods*.

Robotic-assisted surgery is gaining interest in hepatobiliary surgery due to potential advantages such as shorter time to recovery and hospital stay, and a lower morbidity rate as compared with open surgery. No randomized trial concerning robotic-assisted surgery for suspected or confirmed gallbladder cancer has been conducted so far. The present ROBOCOP trial is a multicentre, open label randomized clinical trial aiming to evaluate the robotic-assisted technique and its potential benefits.

## Supplementary Material

zrae168_Supplementary_Data

## Data Availability

Data available on request.
