# Supplementary material for: Robotic-assisted contra open resection for suspected or confirmed gallbladder cancer (ROBOCOP)
Source: BJS Open. 2025 Feb 20;9(1):zrae168. doi: 10.1093/bjsopen/zrae168 (PMC11842188; doi:10.1093/bjsopen/zrae168)
Supplement: Supplementary file 1 [file zrae168_supplementary_data.zip › Supplementary_material.odt]

Robotic-assisted contra open resection for suspected or confirmed gallbladder cancer (ROBOCOP)

Study protocol 

Hannes Wållgren1, Meimene Khalil2, Helena Taflin3, Caroline Williamsson4, Stefan Gilg1, Dennis Björk2, Malin Sternby Eilard3, Peter Strandberg Holka4, Melroy D’Souza1,

Linda Lundgren2, Christian Cahlin3, Bodil Andersson4,  Ernesto Sparrelid1, Per Sandström2,

Niclas Kvarnström3, Magnus Rizell3, Jenny Lundmark Rystedt4, Bergthor Björnsson2,

Christian Sturesson1

1Division of Surgery, Department of Clinical Science, Intervention and Technology (CLINTEC), Karolinska Institutet and Karolinska University Hospital, Stockholm, Sweden.

2Department of Surgery in Linköping and Department of Biomedical and Clinical Sciences, Linköping University, Linköping, Sweden.

3Transplant Institute, Institute of Clinical Sciences, Sahlgrenska Academy at University of Gothenburg, Sahlgrenska University Hospital, Gothenburg, Sweden.

4 Department of Clinical Sciences Lund, Surgery. Lund University and Skåne University Hospital, Lund, Sweden

Corresponding author. Christian Sturesson, Department of surgery, Karolinska University Hospital, 14186 Stockholm, Sweden. Orcid 0000-0003-3451-2840

e-mail: [Christian.sturesson@ki.se](mailto:Christian.sturesson@ki.se) 

Supplementary Materials - Index

| Supplementary Methods |  |
| --- | --- |
| Study design and setting | page 5 |
| Treatment groups Primary endpoint Secondary endpoints Sample size Randomization Implementation Patient follow up Costs Pathology Blood sample collection In-depth analysis of pathology specimens Baseline characteristics Data collection Statistical analysis | page 5 page 6 page 6 page 6 page 7 page 7 page 7 page 7 page 8 page 8 page 8 page 8 page 8 page 9 |

Supplementary Methods

Study design and setting

The ROBOCOP is designed as a multi-centre, open-label, randomized controlled superiority trial. All patients participate within a locally implemented enhanced recovery programme.

Preoperatively, the patients’ baseline characteristics are recorded. Intraoperative and postoperative data is documented during admission and following discharge at 14 days and 3 and 12 months after surgery. The ROBOCOP trial is approved by Swedish Ethical Review Authority (Dnr 2023-01669-01). Patients willing to participate in this trial will receive both verbal and written information at the time of recruitment in the outpatient clinic.

Inclusion criteria: 

- Age above 18 years

- Indication as decided at a multidisciplinary team conference of radical cholecystectomy 

- Patient sufficiently fit to undergo radical cholecystectomy according to surgeon and anaesthesiologist.

- Patient with ASA physical status I, II or III

- Willingness to participate in the study. 

Exclusion criteria:

- Preoperative evidence of tumour invasion of the liver requiring major liver resection, and/or tumour invasion of the hepatoduodenal ligament, and/or tumour invasion of one other organ such as colon, stomach, duodenum 

- Pregnancy

- Inability to provide written informed consent

- Previous extensive open surgery in the upper abdomen (for example open liver surgery).

All patients ineligible for randomisation will be approached for participation in a prospective registry. If patients express an explicit preference for a certain surgical technique, they will be allocated to the prospective registry and interviewed to record the reasons for their preferences.

Treatment groups

Patients fulfilling the inclusion criteria are randomized to the intervention group (robotic-assisted surgery) or control group (open surgery) with the intention of performing radical cholecystectomy, defined here as a wedge resection of the liver comprising the gallbladder fossa or resection of liver segments 4b and 5, and dissection of lymph nodes stations 8 and 12. Routine frozen section of the transection margin of the cystic duct is performed (unless, in case incidental gallbladder cancer found after of previous cholecystectomy and tumour free cystic duct). If a margin positive for cancer is found, further dissection of the cystic duct to achieve a tumour free margin is allowed. 

All patients should be managed within an enhanced recovery protocol. For analgesia, the normal routine of the participating centre concerning open and robotic-assisted analgesia. Patients and ward personnel will not be blinded to the treatment given. Blinding will not be used for the following reasons: the intra- and postoperative analgesic regime includes in some participating centres epidural analgesia for laparotomies and not for minimally invasive procedures. The anaesthetist would therefore have to be aware of the operative approach when discussing with the patient before surgery. Blinding at the ward would be very difficult as the operating surgeons make up a large proportion of the staff taking care of the patients post-operatively. The primary outcome assessment is however done by staff nurses not primarily involved in the study. Patient blinding would also be difficult as Swedish patients have direct access via the internet to their medical charts including the operative report.      

 The surgeon performing the robotic-assisted surgery are required to have performed at least 20 procedures. The senior surgeon in the surgical team performing the open surgery is required to have performed at least 20 procedures.

Surgeons are free to use their preferred technique and surgical devices to gain intra-abdominal access, perform parenchymal transection, maintain vascular control and performing lymph-node dissection. The ports in the robotic-assisted group are placed as preferred by the surgeon. In the open surgery group, access to the liver is through an incision of the surgeon’s choice. There is no standardised technique for liver transection, vascular control and wound closure. 

Primary endpoint

The primary endpoint is time (days) from operation to functional recovery; defined as when the patient

 

- has adequate pain control with oral analgesics only (Numerical Rating Scale<5)

- is independently mobile at the preoperative level

- can maintain sufficient oral caloric intake (minimum of 50% required calories)

- has no need for intravenous fluid administration

- has no clinical signs of infection or infection treated with peroral antibiotics only

The types of oral analgesics to use are not specified, but are recorded. ‘Independently mobile at the preoperative level’ is objectified with a mobility score of 8 or higher (where yes to a question gives one point) according to (13)

Can you fully independently:

- Feed yourself

- Get in and out of bed

- Stand up from sitting in a chair

- Get on and off the toilet

- Wash your face and hands

- Wash and dry your whole body

- Dress yourself 

- Get around in the ward (if necessary, with a cane or walking frame)

- Go up and down the stairs

Secondary endpoints

Secondary endpoints include costs (intra-operative and postoperative direct and indirect costs), quality of life, abdominal wall complaints, number of retrieved lymph nodes, resection margins, length-of-hospital stay (days) and postoperative complications within 90 days of surgery as classified according to the Clavien-Dindo (CD) system with major complications defined as CD IIIa-IV. 

Sample size

Sample size calculation: ROBOCOP is a superiority trial hypothesizing that robotic-assisted surgery results in shorter time to functional recovery compared to open surgery. Time to functional recovery after radical cholecystectomy is not well described in the literature. In the absence of such data, for sample size calculation purposes, hospital length of stay can be used as surrogate for functional recovery. Considering for the patient a similar surgical insult, such as minor liver resections, hospital length of stay after laparoscopic and open resections have been reported to be 2 and 4 days respectively(14). The ROBOCOP trial aims to show a reduction of time to functional recovery of 2 days, which is considered clinically meaningful. A standard deviation of 3.36 days is assumed based on the length of stay for open radical cholecystectomy as calculated from a patient cohort operated at Karolinska University hospital 2019-2020. The significance level (α) is set at 0.05 and power (1-β) at 80%. The sample size needed in each arm to test superiority for the robot-assisted surgery is 35 patients. A 25% drop-out rate is expected including patients undergoing exploration only (in case of intraoperative confirmation of dissemination), patients with intraoperative findings of the need to resect extrahepatic bile ducts and loss to follow up. Therefore, 47 patients will be randomized in each group, with a total of 94 patients to be randomized in the ROBOCOP trial.  

Randomization

Randomisations are performed using a randomisation software (RedCap®, online randomisation module), to create the random allocation sequence we used the Sealed Envelope Ltd. 2022. Block randomisation with permuted block of four (4) and stratified on site. The final list and integration of it to RedCap® were carried out by a third party research assistant. Randomisations are carried out by the local principal investigator, authorised researcher or research nurse. Patient confidentiality is guaranteed by applying pseudo-anonymisation. The patient identification keys are stored locally by each principal investigator.

Implementation

A member of each local research team will be responsible for the randomisation process and will pass the assigned treatment to the treating anaesthesiologist, surgeon, and the operating room staff. Randomization is performed after the patient has given informed consent to participate in the study but before a pre-operative consultation with the anaesthesiologist. The assigned treatment is carried out by the surgeon and the postoperative care is standardised according to the local enhanced recovery after surgery protocol. The surgeon plays no role in postoperative assessments. Postoperative assessment for functional recovery is made by the nursing team or a dedicated research nurse daily in the afternoon between 2-6 pm.  

Patient follow up

Patents will be asked to a complete a validated questionnaire named postoperative recovery profile (PRP)(11) before surgery, at 2 weeks, and 3 and 12 months postoperatively. The PRP has been developed and validated and reliability tested in the context of diverse surgeries1 ADDIN ZOTERO_BIBL {"uncited":[],"omitted":[],"custom":[]} CSL_BIBLIOGRAPHY 1.	Korrel M, Vissers Fl, van Hilst J, de Rooij T, Dijkgraaf Mg, Festen S, et al. Minimally invasive versus open distal pancreatectomy: an individual patient data meta-analysis of two randomized controlled trials. HPB . 2021 Mar;23(3). Available from: https://pubmed.ncbi.nlm.nih.gov/33250330/

2.	Fretland Åa, Dagenborg Vj, Bjørnelv Gmw, Kazaryan Am, Kristiansen R, Fagerland Mw, et al. Laparoscopic Versus Open Resection for Colorectal Liver Metastases: The OSLO-COMET Randomized Controlled Trial. Ann Surg. 2018 Feb;267(2). Available from: https://pubmed.ncbi.nlm.nih.gov/28657937/

3.	Ito H, Ito K, D’Angelica M, Gonen M, Klimstra D, Allen P, et al. Accurate staging for gallbladder cancer: implications for surgical therapy and pathological assessment. Ann Surg. 2011 Aug;254(2). Available from: https://pubmed.ncbi.nlm.nih.gov/21617582/

4.	Sposito C, Ratti F, Cucchetti A, Ardito F, Ruzzenente A, Di Sandro S, et al. Survival benefit of adequate lymphadenectomy in patients undergoing liver resection for clinically node-negative intrahepatic cholangiocarcinoma. J Hepatol. 2023 Feb;78(2). Available from: https://pubmed.ncbi.nlm.nih.gov/36328332/

5.	Yoh T, Cauchy F, Le Roy B, Seo S, Taura K, Hobeika C, et al. Prognostic value of lymphadenectomy for long-term outcomes in node-negative intrahepatic cholangiocarcinoma: A multicenter study. Surgery. 2019 Dec;166(6). Available from: https://pubmed.ncbi.nlm.nih.gov/31421869/

6.	Aloia Ta, Járufe N, Javle M, Maithel Sk, Roa Jc, Adsay V, et al. Gallbladder cancer: expert consensus statement. HPB. 2015 Aug;17(8). Available from: https://pubmed.ncbi.nlm.nih.gov/26172135/

7.	Ratti F, Cipriani F, Ingallinella S, Tudisco A, Catena M, Aldrighetti L. Robotic Approach for Lymphadenectomy in Biliary Tumors: The Missing Ring Between the Benefits of Laparoscopic and Reproducibility of Open Approach. Ann Surg. 2023 Oct 1;278(4):e780–8. 

8.	Jiayi W, Shelat V. Robot-assisted radical cholecystectomy for gallbladder cancer: A review. J Clin Transl Res. 2022 Feb 2;8:103–9. 

9.	Kone Lb, Bystrom Pv, Maker Av. Robotic Surgery for Biliary Tract Cancer. Cancers. 2022 Feb 18;14(4). Available from: https://pubmed.ncbi.nlm.nih.gov/35205792/

10.	Schulz Kf, Altman Dg, Moher D. CONSORT 2010 Statement: updated guidelines for reporting parallel group randomised trials. BMC Med. 2010 Mar 24;8. Available from: https://pubmed.ncbi.nlm.nih.gov/20334633/

11.	Allvin R, Ehnfors M, Rawal N, Svensson E, Idvall E. Development of a questionnaire to measure patient-reported postoperative recovery: content validity and intra-patient reliability. J Eval Clin Pract. 2009 Jun;15(3). Available from: https://pubmed.ncbi.nlm.nih.gov/19366398/

12.	Clay L, Fränneby U, Sandblom G, Gunnarsson U, Strigård K. Validation of a questionnaire for the assessment of pain following ventral hernia repair--the VHPQ. Langenbecks Arch Surg. 2012 Dec;397(8). Available from: https://pubmed.ncbi.nlm.nih.gov/22446989/

13.	Suurmeijer Tp, Doeglas Dm, Moum T, Briançon S, Krol B, Sanderman R, et al. The Groningen Activity Restriction Scale for measuring disability: its utility in international comparisons. Am J Public Health. 1994 Aug;84(8). Available from: https://pubmed.ncbi.nlm.nih.gov/8059884/

14.	Schultz Na, Larsen Pn, Klarskov B, Plum Lm, Frederiksen Hj, Kehlet H, et al. Second Generation of a Fast-track Liver Resection Programme. World J Surg. 2018 Jun;42(6). Available from: https://pubmed.ncbi.nlm.nih.gov/29302723/

15.	Ayabe Ri, Azimuddin A, Tran Cao Hs. Robot-assisted liver resection: the real benefit so far. Langenbecks Arch Surg. 2022 Aug;407(5). Available from: https://pubmed.ncbi.nlm.nih.gov/35488913/

16.	Huang L, Zhang C, Tian Y, Liao C, Yan M, Qiu F, et al. Laparoscopic segment 4b+5 liver resection for stage T3 gallbladder cancer. Surg Endosc. 2022 Dec [cited 2024 Feb 6];36(12). Available from: https://pubmed.ncbi.nlm.nih.gov/35906460/

. In addition, an abdominal wall-specific questionnaire will be used; the validated ventral hernia pain questionnaire (VHPQ)(12). It consists of 19 questions that reflect the patients’ perception of the incisional site concerning pain, cosmetic issues, social limitations, and abdominal wall stiffness. Also, questions concerning the level, frequency and duration of pain, use of pain medication and impact on daily activities are included. Clinically significant symptoms are determined as: pain right now/last week-not easily ignored, scar cosmetically disturbing, scar socially limiting and abdominal wall stiffness. The patients will be asked to complete the VHPQ at baseline, at 2 weeks, and 3, and 12 months postoperatively. 

Patients with proven gallbladder cancer will be followed according to regional guidelines and cancer-specific survival will be calculated after 3 years stratified according to operation technique and TN-stage. For patients with an indication of adjuvant chemotherapy, the time to initiation of chemotherapy is recorded as well as the number of intended chemotherapy cycles administered.   

Quality of life is measured by the EQ-5D-5L and EORTC QLQ C-30 questionnaires. 

Costs 

The present study will analyse and compare direct costs, comprising all costs related to the treatment and patient care. Indirect costs defined as costs related to loss of productivity will also be analysed although the age of a significant number of patients can be assumed to be higher than their retirement age. Direct costs will be estimated for the procedures, hospital stay and follow up. The procedural period includes all events from admission to discharge and comprises costs of radiological imaging, procedure-related (anaesthesia, operating room time, consumable supplies, staff salary), daily hospitalization fee (medications, intravenous solutions, laboratory testing and staff salary). Costs applied to the follow-up period include costs of appointments to the surgical outpatient clinic, emergency-room visit as well as cost for hospital readmission during a 3-month period after surgery. 

   

Pathology

The number of analysed lymph-nodes are reported. If the surgical procedure encompassed lymph-nodes beyond the stations 8 and 12 (due to intraoperative suspicion of tumour invasion), these lymph-node stations are accounted for separately. If gallbladder cancer is found, the minimal distance from the tumour border to the resected liver parenchyma is measured and denoted oncological gallbladder resection margin. In patients re-resected after incidentally discovered gallbladder cancer after cholecystectomy, margin is only measured in patients were residual cancer cells are found during pathology microscopy exam, and is then denoted oncological margin, and is analysed together with patients with a measured oncological gallbladder resection margin as described above. For patients with gallbladder cancer, resection margins are classified as R0 shortest margin ≥1 mm (microscopically radical), R1 shortest margin < 1 mm (microscopically involved) and R2 no margin (macroscopically visible tumour on the cut surface).

Blood sample collection

Blood samples will be taken longitudinally before and after surgery. Two options for blood sample collection will be available: basic and extended protocol. For the basic protocol: 4ml serum tube + 4ml EDTA tube will be taken preoperative (same day or day before surgery) and 24 hours after surgery (two timepoints). For the extended protocol: 4ml serum tube + 4ml EDTA tube + two 8ml (16ml) heparin tubes will be taken preoperative (same day or day before surgery), 4-6 hours after start of surgery, 24 hours after surgery, and at the first postoperative outpatient visit (typically 4-6 weeks after surgery) (four timepoints in total).

In-depth analysis of pathology specimens

After regular histopathological assessment has been performed, a request to scan, digitalize, and share available histopathology slides from included patients will be sent to the respective clinical pathology department. This will allow for coordinated in-depth analysis of the GBC tumormicroenvironment (TME) including usage of AI-algorithms. Furthermore, a request will be made to each clinical pathology department for acquisition of additional sections (up to 15) from each patient to facilitate more in-depth analysis of the TME.

Baseline characteristics

Baseline criteria are ascertained on the day of admission to the hospital. These include age, sex, social situation (living alone, with relatives or partner, or at a community centre), body mass index, preoperative medication, disease characteristics, preoperative treatment if applicable and prior abdominal surgery. Preoperatively, patient’s comorbidities are registered descriptively and summarised with the American Society of Anaesthesiologists (ASA) physical status classification system. Venous blood samples are drawn and analysed for blood group, haemoglobin, leucocytes, platelets, activated partial thromboplastin time, international normalised ratio, creatinine, liver function tests (ASAT, ALAT, Bilirubin, ALP), C-reactive protein and if applicable carcinoembryonic antigen.

Data collection

All patient data are prospectively collected either on paper case record forms (CRFs) or directly entered into eCRFs on RedCap®. All data are stored in compliance with good clinical practice guidelines on a European Union-based server, referring to the patient’s trial identification code (created by the randomisation software) to ensure patient confidentiality. Local researchers have access to their site data. Only the principal and coordinating investigator and the trial statistician have access to the final dataset. The trial coordinator will conduct regular audits regarding data completeness and plausibility. Data is kept for 10 years after completion of the trial.

Statistical analysis

The primary hypothesis of the ROBOCOP trial is that robotic-assisted radical cholecystectomy is superior to an open approach in terms of short-term surgical outcomes. To assess the hypothesis, patients are analysed by a per protocol approach: all randomised patients with available study data and who do not withdraw consent are analysed according to their treatment allocation, unless post-randomization events as presented below occur:

Intraoperative findings of dissemination (patient data is then analysed according to intention-to-treat).

Intraoperative findings of the need to perform resection of extrahepatic bile ducts (patient data is then analysed according to intention-to-treat).

Intraoperative findings leading to a simple cholecystectomy only (patient data is then analysed according to intention-to-treat).

Patients for whom the surgery was converted (that is, changed intraoperatively from robotic-assisted to open) remain within the robotic-assisted group for all analyses, as conversion is an intrinsic risk of robotic-assisted surgery. Baseline characteristics are presented as means and standard deviations or as medians and interquartile range, where appropriate. Standardised mean differences are explored. Time to functional recovery is measured in days and is analysed using fixed effects linear regression, considering treatment, patient age and sex as covariates. 

All other outcomes are similarly analysed with regression (linear for quantitative outcomes, logistic for binary outcomes), applying a two-tailed alpha = 0.01 to adjust for multiple testing. Repeatedly measured outcomes are analysed with mixed regression for repeated measures to include participants with missing data. Proper and accepted methods are used for handling missing data.

References

1.	Korrel M, Vissers Fl, van Hilst J, de Rooij T, Dijkgraaf Mg, Festen S, et al. Minimally invasive versus open distal pancreatectomy: an individual patient data meta-analysis of two randomized controlled trials. HPB . 2021 Mar;23(3). Available from: https://pubmed.ncbi.nlm.nih.gov/33250330/

2.	Fretland Åa, Dagenborg Vj, Bjørnelv Gmw, Kazaryan Am, Kristiansen R, Fagerland Mw, et al. Laparoscopic Versus Open Resection for Colorectal Liver Metastases: The OSLO-COMET Randomized Controlled Trial. Ann Surg. 2018 Feb;267(2). Available from: https://pubmed.ncbi.nlm.nih.gov/28657937/

3.	Ito H, Ito K, D’Angelica M, Gonen M, Klimstra D, Allen P, et al. Accurate staging for gallbladder cancer: implications for surgical therapy and pathological assessment. Ann Surg. 2011 Aug;254(2). Available from: https://pubmed.ncbi.nlm.nih.gov/21617582/

4.	Sposito C, Ratti F, Cucchetti A, Ardito F, Ruzzenente A, Di Sandro S, et al. Survival benefit of adequate lymphadenectomy in patients undergoing liver resection for clinically node-negative intrahepatic cholangiocarcinoma. J Hepatol. 2023 Feb;78(2). Available from: https://pubmed.ncbi.nlm.nih.gov/36328332/

5.	Yoh T, Cauchy F, Le Roy B, Seo S, Taura K, Hobeika C, et al. Prognostic value of lymphadenectomy for long-term outcomes in node-negative intrahepatic cholangiocarcinoma: A multicenter study. Surgery. 2019 Dec;166(6). Available from: https://pubmed.ncbi.nlm.nih.gov/31421869/

6.	Aloia Ta, Járufe N, Javle M, Maithel Sk, Roa Jc, Adsay V, et al. Gallbladder cancer: expert consensus statement. HPB. 2015 Aug;17(8). Available from: https://pubmed.ncbi.nlm.nih.gov/26172135/

7.	Ratti F, Cipriani F, Ingallinella S, Tudisco A, Catena M, Aldrighetti L. Robotic Approach for Lymphadenectomy in Biliary Tumors: The Missing Ring Between the Benefits of Laparoscopic and Reproducibility of Open Approach. Ann Surg. 2023 Oct 1;278(4):e780–8. 

8.	Jiayi W, Shelat V. Robot-assisted radical cholecystectomy for gallbladder cancer: A review. J Clin Transl Res. 2022 Feb 2;8:103–9. 

9.	Kone Lb, Bystrom Pv, Maker Av. Robotic Surgery for Biliary Tract Cancer. Cancers. 2022 Feb 18;14(4). Available from: https://pubmed.ncbi.nlm.nih.gov/35205792/

10.	Schulz Kf, Altman Dg, Moher D. CONSORT 2010 Statement: updated guidelines for reporting parallel group randomised trials. BMC Med. 2010 Mar 24;8. Available from: https://pubmed.ncbi.nlm.nih.gov/20334633/

11.	Allvin R, Ehnfors M, Rawal N, Svensson E, Idvall E. Development of a questionnaire to measure patient-reported postoperative recovery: content validity and intra-patient reliability. J Eval Clin Pract. 2009 Jun;15(3). Available from: https://pubmed.ncbi.nlm.nih.gov/19366398/

12.	Clay L, Fränneby U, Sandblom G, Gunnarsson U, Strigård K. Validation of a questionnaire for the assessment of pain following ventral hernia repair--the VHPQ. Langenbecks Arch Surg. 2012 Dec;397(8). Available from: https://pubmed.ncbi.nlm.nih.gov/22446989/

13.	Suurmeijer Tp, Doeglas Dm, Moum T, Briançon S, Krol B, Sanderman R, et al. The Groningen Activity Restriction Scale for measuring disability: its utility in international comparisons. Am J Public Health. 1994 Aug;84(8). Available from: https://pubmed.ncbi.nlm.nih.gov/8059884/

14.	Schultz Na, Larsen Pn, Klarskov B, Plum Lm, Frederiksen Hj, Kehlet H, et al. Second Generation of a Fast-track Liver Resection Programme. World J Surg. 2018 Jun;42(6). Available from: https://pubmed.ncbi.nlm.nih.gov/29302723/

15.	Ayabe Ri, Azimuddin A, Tran Cao Hs. Robot-assisted liver resection: the real benefit so far. Langenbecks Arch Surg. 2022 Aug;407(5). Available from: https://pubmed.ncbi.nlm.nih.gov/35488913/

16.	Huang L, Zhang C, Tian Y, Liao C, Yan M, Qiu F, et al. Laparoscopic segment 4b+5 liver resection for stage T3 gallbladder cancer. Surg Endosc. 2022 Dec [cited 2024 Feb 6];36(12). Available from: https://pubmed.ncbi.nlm.nih.gov/35906460/
